# Chemogenetic hyperpolarization in the entorhinal cortex during sleep facilitates delta oscillations and memory consolidation

**DOI:** 10.1016/j.isci.2026.118120

**Published:** 2026-11-05

**Authors:** Suman Gunin, Judy Vu, Juhee Haam

**Affiliations:** 1 Department of Biological Sciences, Louisiana State University, Baton Rouge, LA 70803, USA

**Keywords:** chemogenetics, memory consolidation, entorhinal cortex, delta oscillations, temporoammonic pathway, photometry, sleep, hM3Dq, hM4Di, and behavior

## Abstract

Memory consolidation is a crucial step in long-term memory formation, during which stable memory representations are formed using recently encoded labile memories. The entorhinal cortex (EC) mediates such brain-wide memory reorganization by exhibiting delta oscillations during sleep, through hyperpolarization-activated cyclic nucleotide-gated (HCN) channels. We tested the feasibility of chemogenetically manipulating EC delta oscillations and examined whether such manipulations affect memory consolidation. We demonstrate that membrane hyperpolarization via the activation of the inhibitory chemogenetic receptor hM4Di paradoxically facilitates delta oscillations in the EC by activating HCN channels, whereas membrane depolarization via the activation of the excitatory chemogenetic receptor hM3Dq induces the opposite effect. Furthermore, we show that chemogenetic depolarization during sleep following learning impairs memory consolidation, whereas chemogenetic hyperpolarization enhances it. Our study provides insights into the effects of chemogenetic manipulation on neural oscillations and identifies EC delta as a key regulator of memory consolidation efficiency.

## Highlights

• Chemogenetic depolarization attenuates entorhinal cortical delta during sleep
• Chemogenetic hyperpolarization augments entorhinal cortical delta during sleep
• Chemogenetic depolarization during sleep impairs memory consolidation
• Chemogenetic hyperpolarization during sleep enhances memory consolidation

## Graphical abstract

**Figure:**
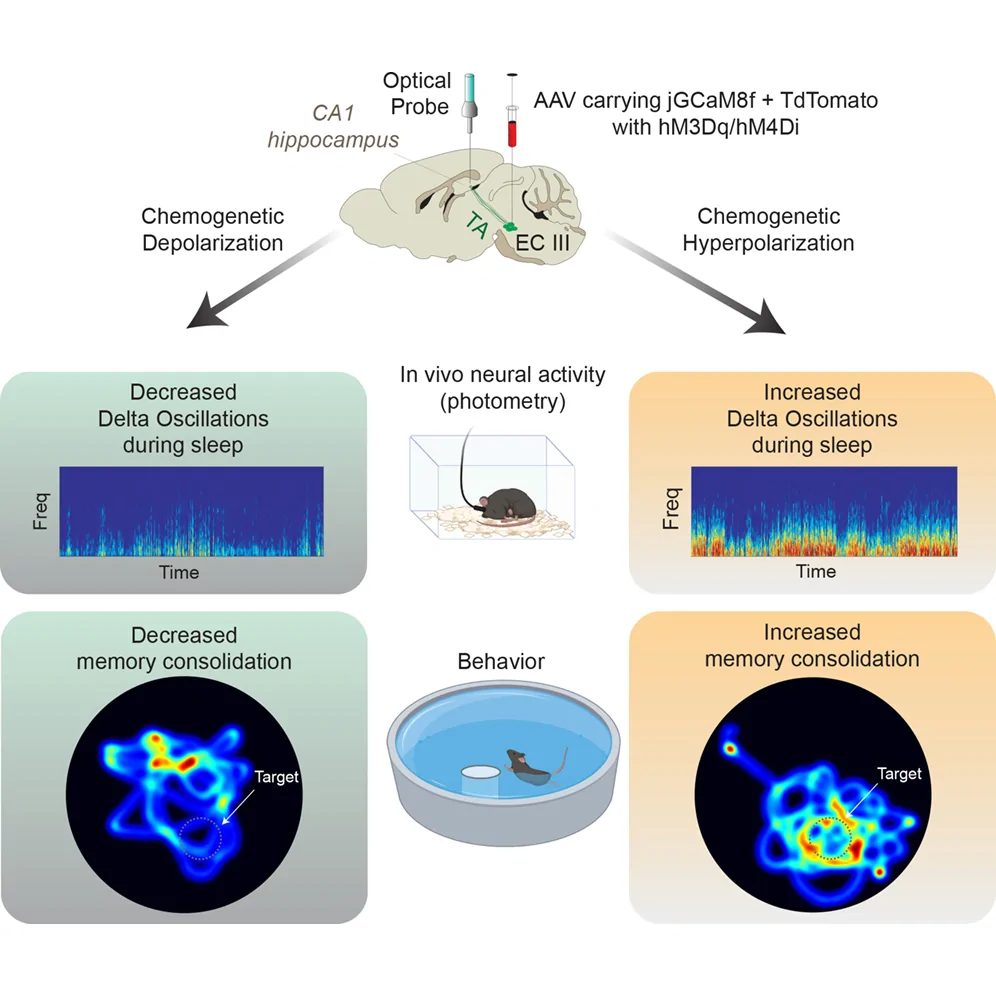

## Teaser

Behavioral neuroscience; Molecular neuroscience; Cellular neuroscience

## Introduction

Memory consolidation is the process of forming long-lasting, stable memory representations in the brain using newly encoded memory representations in the hippocampus, which are labile and prone to decay.^1^^,^^2^ During non-rapid eye movement (NREM) sleep following learning, these hippocampal neuronal representations are reactivated, leading to the formation of widely distributed memory ensembles in the neocortex, via a process known as systems consolidation.^1^^,^^3^^,^^4^^,^^5^

The entorhinal cortex (EC), with extensive connections to the hippocampus and neocortex, serves as an information hub between these brain structures.^6^^,^^7^^,^^8^ The temporoammonic (TA) pathway in layer III of the entorhinal cortex (ECIII) provides a monosynaptic projection to the hippocampal CA1 region, where encoded memories are stored and the major hippocampal output neurons are located.^9^^,^^10^ Neural oscillations are critical components of systems consolidation by mediating large-scale communication between the hippocampus and the neocortex.^4^^,^^11^^,^^12^^,^^13^^,^^14^ Our previous work identified delta oscillations exhibited by TA pathway neurons in the EC via hyperpolarization-activated cyclic nucleotide-gated (HCN) channels during NREM sleep and demonstrated that these channel activities are crucial for memory consolidation.^15^ HCN channels are activated by a negative shift in membrane potential (i.e., membrane hyperpolarization), and upon activation, they depolarize the membrane due to their permeability to cations. The HCN channels’ self-oscillatory property underlies pacemaking activities of thalamic neurons in the brain and in cardiac cells of the sinoatrial node of the heart.^16^^,^^17^ Given that HCN channels are activated by membrane hyperpolarization, the remaining important question is whether membrane hyperpolarization in the TA pathway would facilitate delta oscillations and memory consolidation or would suppress the consolidation process due to a reduction in spiking activity.

For the manipulation of neuronal activity, one of the commonly used approaches is chemogenetics, which utilizes genetically expressed engineered receptors that are only activated by synthetic ligand molecules.^18^^,^^19^^,^^20^ The chemogenetic receptors hM3Dq and hM4Di are engineered human muscarinic acetylcholine receptors. Upon activation by the synthetic ligand deschloroclozapine (DCZ), hM3Dq, a G_q_-coupled receptor, results in membrane depolarization through the activation of phospholipase-dependent signaling,^18^^,^^19^ whereas hM4Di, a G_i_-coupled receptor, induces membrane hyperpolarization via the activation of G protein-coupled inwardly rectifying potassium (GIRK) channels. Notably, three important points are to be considered regarding the effect of chemogenetic receptor activation on delta oscillations in the TA pathway: 1) A change in spiking activity would occur with membrane depolarization and hyperpolarization, affecting the power of oscillations; 2) Chemogenetic membrane hyperpolarization can cause the activation of HCN channels in the TA pathway, leading to subsequent membrane depolarization and rhythmic activity of these neurons; however, the effect on oscillations depends on the resting membrane potential and basal levels of HCN channel activation before the chemogenetic manipulation. Similarly, the effect of membrane depolarization on delta oscillations depends on the endogenous level of HCN channel activity; 3) the degree of chemogenetic membrane hyperpolarization relies on GIRK channel expression in TA pathway neurons, whereas the degree of chemogenetic membrane depolarization is dependent on the endogenous levels of M-current, which is a non-inactivating potassium current. Considering these points, the overall effects of hM4Di and hM3Dq receptor activation on delta oscillations can only be experimentally determined.

We employed cell-type- and input-specific recordings of TA pathway neurons in naturally behaving animals using *in vivo* fiber photometry.^15^ We found that chemogenetic membrane hyperpolarization, while typically considered as “neuronal inhibition”, induces a significant increase in delta oscillations in TA pathway neurons. On the other hand, chemogenetic membrane depolarization significantly reduced delta oscillations in the TA pathway. Using rodent behavioral testing, we demonstrate that chemogenetic depolarization during sleep immediately following new learning reduces memory consolidation, whereas chemogenetic hyperpolarization enhances it. Thus, our study demonstrates that membrane potential manipulation modulates memory consolidation in a manner that parallels its effect on neural oscillations but opposes its change in spiking activity.

## Results

### Chemogenetic control of membrane potential modulates delta oscillations in the TA pathway

To investigate the effect of membrane potential modulation on delta oscillations in the TA pathway, we employed the chemogenetic approach. The rationale for selecting chemogenetics rather than optogenetics was that this experiment required sustained modulation over a longer timescale to investigate its effect on neural oscillations and behavior. Prolonged activation of inhibitory opsins is shown to cause altered ionic concentrations, inducing paradoxical excitatory effects.^21^^,^^22^^,^^23^^,^^24^ Furthermore, the light illumination required for optogenetics in the target brain region could interfere with photometry recordings from the same region. Thus, we employed a chemogenetic approach to modulate neuronal membrane potential in the EC. Given that HCN channels drive delta oscillations in the EC and that their gating depends on membrane hyperpolarization, we reasoned that activating hM4Di would significantly facilitate delta activity, whereas activating hM3Dq would have a suppressive effect.

We first examined the specificity of the chemogenetic receptor expression using immunohistochemistry. Mice were injected with adeno-associated virus (AAV) carrying the hM3Dq gene or the hM4Di gene, glutamatergic neuron-specific, *Camk2a* promoter (AAV-CaMKIIα-hM3Dq-mCherry or AAV-CaMKIIα-hM4Di-mCherry) in layer III of EC (ECIII). Six to 12 weeks after viral injection, brain tissues were collected for immunostaining. Vesicular glutamate transporter 1 (VGLUT1), the predominant vesicular glutamate transporter expressed in the EC, is primarily localized to axon terminals, making the identification of glutamatergic neurons extremely challenging by immunoreactivity. Thus, we used the GABAergic marker glutamic acid decarboxylase (GAD65 and GAD67) along with the neuronal marker NeuN to identify putative excitatory neurons. In the caudomedial EC, where the virus containing chemogenetic receptors was injected, 99.5% (997 out of 1002 cells) of mCherry-positive hM3Dq-expressing cells were immunopositive to NeuN and immunonegative to GAD65 and GAD67 (Figure S1C), suggesting that hM3Dq was primarily expressed in excitatory neurons. Likewise, 99.5% (1013 out of 1018 cells) of mCherry-positive hM4Di-expressing cells were NeuN-immunopositive and GAD67-immunonegative (Figure S1D). Among the putative excitatory neurons, which are NeuN-positive but GAD67-negative, 62.8% (1013 out of 1611 cells) and 60.2% (997 out of 1655 cells) were positive for mCherry-hM3Dq (Figure S1E) and mCherry-hM4Di (Figure S1F), respectively.

To investigate changes in membrane potential induced by the chemogenetic receptors, we used *ex vivo* electrophysiology. Four weeks after mice were injected with AAV-CaMKIIα-hM3Dq-mCherry in ECIII, brain sections were prepared for patch-clamp recordings. DCZ (500 nM) was applied via a puff electrode (5–10 psi for 2 s). In mCherry-positive hM3Dq-expressing cells, puff application of DCZ caused a positive shift in the membrane potential (Figures S1G–S1I). On the other hand, in hM3Dq-negative cells, identified by a lack of mCherry expression, puff application of DCZ caused no significant change in the membrane potential (Figures S1G–S1I), demonstrating the specificity of chemogenetic modulation.

To specifically record neural oscillations of TA pathway neurons in freely moving animals, we employed an approach combining genetic expression of a calcium indicator with pathway-specific fiber photometry recordings, which we previously developed.^15^ TA pathway neurons are glutamatergic neurons that project from ECIII to CA1 of the hippocampus.^6^^,^^25^ The cocktail of AAVs containing the calcium indicator GCaMP6f or jGCaMP8f and tdTomato genes under the CaMKIIα promoter (AAV-CaMKIIα-GCaMP6f-WPRE or AAV-CaMKIIα-jGCaMP8f-WPRE and AAV-CaMKIIα-tdTomato-WPRE) was injected into the ECIII of adult male mice (Figures 1A and S2). Optical probes targeting the terminals of GCaMP-expressing TA pathway neurons were implanted in the CA1 area of the hippocampus. This approach, which utilizes cell-type-specific expression of the calcium indicator and terminal-targeting recordings, allowed the isolation of TA pathway neurons not only from GABAergic neurons but also from local glutamatergic neurons in the EC. To eliminate movement artifacts, GCaMP signals were normalized to the tdTomato signal.^15^^,^^26^^,^^27^ All these mice also received AAV carrying a chemogenetic actuator (AAV-CaMKIIα-hM3Dq-mCherry or AAV-CaMKIIα-hM4Di-mCherry) in the ECIII for chemogenetic modulation of TA pathway neurons (Figure 1A). We performed 2-h *in vivo* photometry recordings during the early phase of the light cycle, when EC delta activity is predominant. To determine the effect of chemogenetic modulation, we conducted recordings over four consecutive days under four conditions: baseline, saline, DCZ (0.1 mg/kg), and washout.

**Figure 1:**
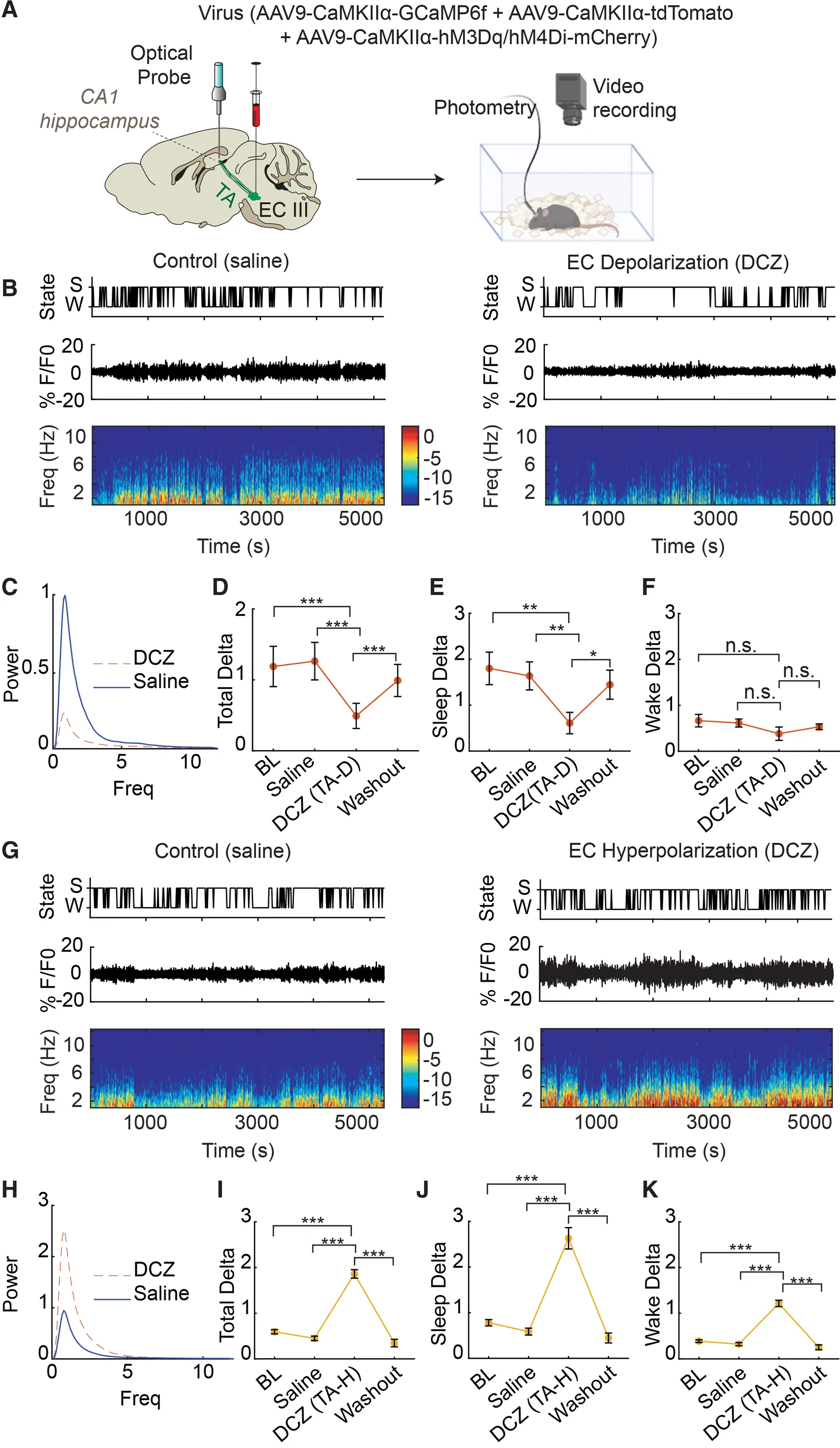
Chemogenetic manipulation of membrane potential effectively modulates delta oscillations in the TA pathway during sleep (A) Schematic shows the injection of AAVs carrying the GCaMP6f, tdTomato, and a chemogenetic actuator (hM3Dq or hM4Di) genes under the glutamatergic-neuron-specific promoter CaMKIIα into ECIII and the implantation of optical probes in the CA1 area targeting TA pathway terminals. Photometry recordings of the TA pathway were done simultaneously with video recordings in the mice’s home cage during the light phase. (B) Representative machine-learning-based sleep/wake classification (top), filtered photometry recording trace (middle), and spectrogram of the photometry signals (bottom) from an hM3Dq-injected mouse after saline and DCZ injection. (C) The power spectral density function of photometry signals after saline injection (solid blue) or hM3Dq activation (dashed red). (D–F) Summary graph shows the power of delta during the entire recording (D), during the sleep state (E), and during the waking state (F) over the subsequent recordings of baseline, saline, DCZ, and washout in hM3Dq-expressing mice (*n* = 4). Repeated measures One-way ANOVA followed by Holm-corrected post-hoc comparisons for Total Delta, baseline vs. DCZ, *p* < 0.001, saline vs. DCZ, *p* < 0.001, DCZ vs. washout, *p* < 0.001; Sleep Delta, baseline vs. DCZ, *p* = 0.003, saline vs. DCZ, *p* = 0.006, DCZ vs. washout, *p* = 0.017; Wake Delta, baseline vs. DCZ, *p* = 0.304, saline vs. DCZ, *p* = 0.489, DCZ vs. washout, *p* = 1.000. (G) Representative sleep/wake classification (top), filtered photometry recording trace (middle), and spectrogram from the photometry signals (bottom) from a hM4Di-injected mouse after saline and DCZ injection. (H) The power spectral density function of photometry signals after saline injection (solid blue) or hM4Di activation (dashed red). (I–K) Summary graph shows the power of delta during the entire recording (I), during the sleep state (J), and during the waking state (K) over the subsequent recordings of baseline, saline, DCZ, and washout in hM4Di-expressing mice (*n* = 5). Repeated measures One-way ANOVA followed by Holm-corrected post-hoc comparisons for Total Delta, baseline vs. DCZ, *p* < 0.001, saline vs. DCZ, *p* < 0.001; DCZ vs. washout, *p* < 0.001; Sleep Delta, baseline vs. DCZ, *p* < 0.001; saline vs. DCZ, *p* < 0.001, DCZ vs. washout, *p* < 0.001; Wake Delta, baseline vs. DCZ, *p* < 0.001; saline vs. DCZ, *p* < 0.001, DCZ vs. washout, *p* < 0.001. Data are presented as mean ± SEM. The reported sample sizes correspond to the number of mice. n.s. *p* > 0.05, ∗*p* < 0.05, ∗∗*p* < 0.01, and ∗∗∗*p* < 0.001.

In mice with hM3Dq expression in the EC, photometry recordings of the TA pathway during baseline and saline sessions (days 1 and 2), without any agonist of hM3Dq, showed potent delta oscillations, similar to those in mice without chemogenetic receptor expression, as we previously reported.^15^ The filtered, normalized signals showed elevated oscillatory activity during sleep bouts, and spectral analysis revealed prominent delta oscillations during sleep (Figure 1B). Interestingly, activation of hM3Dq with DCZ administration (Day 3) caused a significant reduction in oscillatory activity and average delta power over 2-h recordings compared to the baseline and saline recordings (F(3,9) = 8.288, *p* = 0.006; Figures 1B–1D). This reduction was reversible, with the next day of recording without treatment (washout) (Day 4) showing comparable levels of delta activity to the baseline and saline recordings.

Chemogenetic depolarization of the EC had no significant effect on sleep amount, sleep bout duration, and sleep bout numbers, suggesting that the reduction in delta by membrane depolarization was not due to a change in sleep architecture (Figure S4). Furthermore, analysis with sleep state classification indicated that the reduction in the average delta power caused by DCZ activation of hM3Dq is attributed to the reduction in delta power during the sleep state (F(3,9) = 11.497, *p* = 0.002), without affecting delta oscillations during waking (F(3,9) = 1.939, *p* = 0.194; Figures 1E and 1F). These results suggest that hM3Dq-induced membrane depolarization reduces delta oscillations during sleep, which may be due to reduced activation of HCN channels.

Next, we sought to determine whether chemogenetic hyperpolarization of EC neurons would increase delta oscillations via the increased activation of HCN channels. In hM4Di-expressing mice, DCZ activation of these receptors (day 3) caused an increase in average delta power compared to the baseline and saline recordings (F(3,12) = 81.443, *p* < 0.001; Figures 1G–1I). Further analysis of sleep classification demonstrated that DCZ activation of hM4Di caused a significant increase in delta power during sleep (F(3,12) = 40.689, *p* < 0.001; Figure 1J). However, unlike hM3Dq activation, activation of hM4Di also caused a significant increase in delta during waking, even though the magnitude of the change was smaller than during sleep. These results suggest that hM4Di-induced membrane hyperpolarization facilitates HCN channel activation to such an extent that delta oscillations are generated even in the waking state (F(3,12) = 67.320, *p* < 0.001; Figure 1K). These results also suggest that endogenous levels of HCN channel activation are not at full capacity and can be further excited upon membrane hyperpolarization. Chemogenetic depolarization and hyperpolarization of EC had no significant effect on theta oscillations (Figure S3), suggesting that the effect is selective to delta oscillations.

### Chemogenetic modulation of the membrane potential of the TA pathway remodels the consolidation efficacy of spatial memory

As we have previously shown that delta oscillations in the TA pathway drive memory consolidation during sleep,^15^ we reasoned that chemogenetic modulation of TA delta activity would enhance or disrupt memory consolidation. Thus, we investigated the effect of chemogenetic modulation on memory consolidation using the Morris water maze (MWM), a well-established rodent behavioral test for measuring spatial memory.^28^^,^^29^ During the training phase, mice navigate an open swimming arena using distal extramaze cues to locate a submerged escape platform, and the acquisition of spatial memory was assessed by the time required for the mice to locate the platform, known as escape latency. The consolidation of spatial memory from the training phase was assessed in two probe trials, which occurred one day and eight days after the last training day. During the probe sessions, the hidden platform was removed, and the percentage of time spent in each quadrant was measured (Figure 2A). To investigate the effect of chemogenetic modulation of the TA pathway on memory consolidation, adult mice were injected with AAV carrying either the hM3Dq or hM4Di under the glutamatergic neuron-specific promoter CaMKIIα (AAV-CaMKIIα-hM3Dq-mCherry or CaMKIIα-hM4Di-mCherry) into ECIII at least four weeks before the MWM task. The control group received AAV carrying a fluorophore gene (AAV9-CaMKIIα-tdTomato or AAV9-hSyn-DIO-mCherry). DCZ (0.1 mg/kg) was administered intraperitoneally (i.p.) to all three groups of mice immediately after the training sessions on the second and third training days, while the first training day was used to establish baseline performance for all three groups (Figure 2A). All training sessions occurred at the end of the dark cycle, and chemogenetic manipulation began during the light phase, when the majority of sleep occurs in rodents. Compared to the control mice, mice with chemogenetic depolarization (CaMKIIα-hM3Dq-mCherry) and hyperpolarization (CaMKIIα-hM4Di-mCherry) of EC showed similar levels of decrease in escape latency over the training days (F(2,25) = 2.147, *p* = 0.138; Figure 2B). The daily learning rate, calculated from the difference between escape latency in the last block and in the first block of the day, showed comparable learning across all three groups (Figures S5A–S5C). As Day 3 training occurs after DCZ injection, we also examined in detail whether any trial performance during this training differs among groups. Trial-based analysis showed no significant difference in escape latency among the three groups on Day 3 (Figure S5D). In addition, we investigated whether chemogenetic modulation differentially affects the learning rates across blocks on Day 3; the EC-hyperpolarization and depolarization groups did not differ significantly from the control group in block-wise learning rate on Day 3 (Learning rate from the first to second block, F(2,25) = 0.548, *p* = 0.585; learning rate from second to third block, F(2,25) = 0.200, *p* = 0.820; Figures S5E and S5F). These data suggest that chemogenetic modulation has no effect on the acquisition of spatial memory during the training phase.

**Figure 2:**
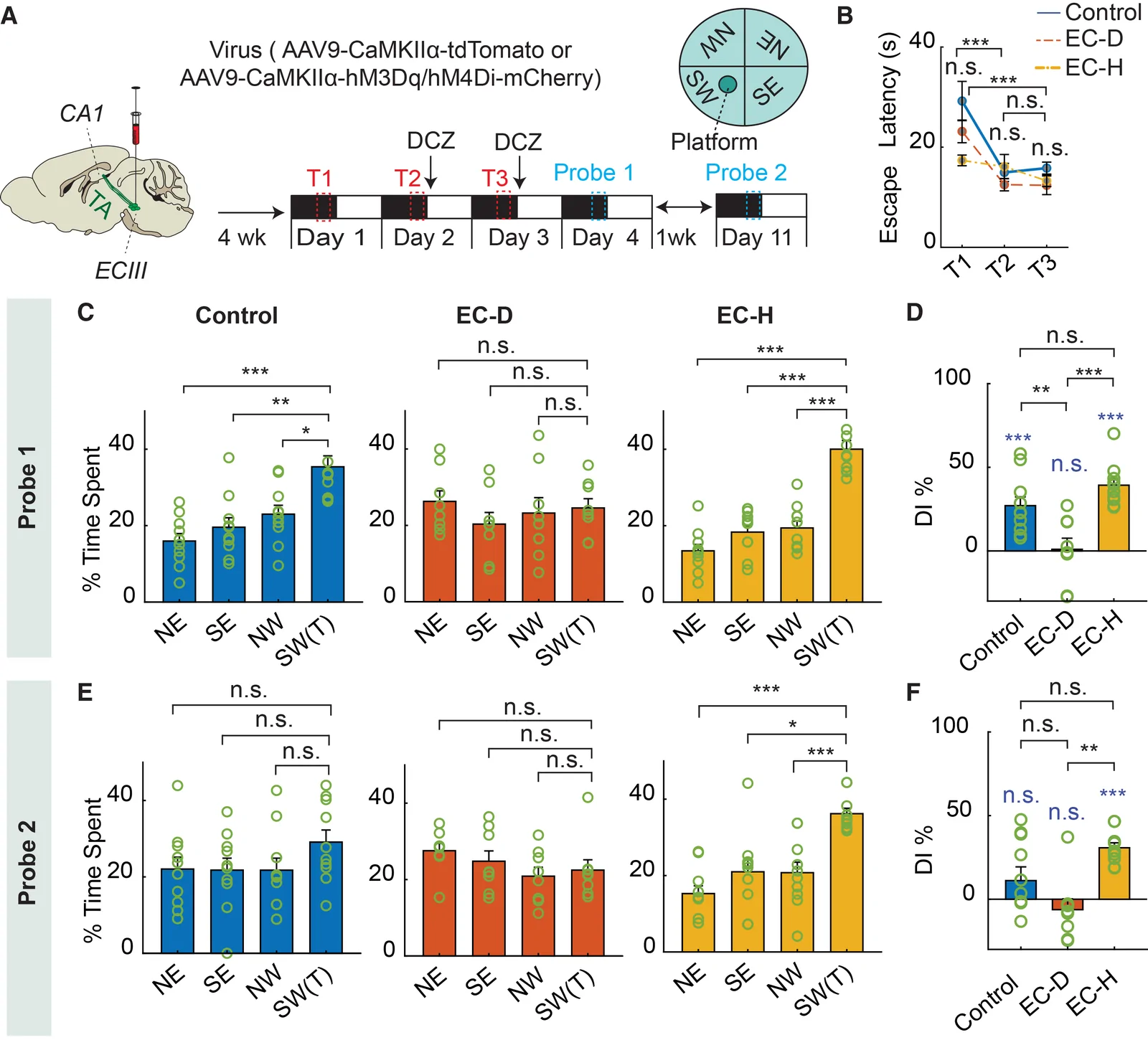
The consolidation of spatial memory is impaired by chemogenetic depolarization of EC during sleep following training, while enhanced by chemogenetic hyperpolarization (A) Images show the timeline of the Morris water maze (MWM) task, and the platform and quadrants used for the task. (B) Summary graph shows the average escape latency over training days. The spatial memory acquisition of mice with chemogenetic depolarization (red dashed line) and hyperpolarization (orange dash-dot-dash line) of the TA pathway was not significantly different from that of control mice (blue solid line) during the training phase. Two-way mixed ANOVA followed by Holm-corrected post-hoc comparison among the three training days. Day 1 vs. Day 2, *p* < 0.001; Day 1 vs. Day 3, *p* < 0.001; Day 2 vs. Day 3, *p* = 0.634. Post-hoc comparison between subject factors. Day1: control vs. EC-depolarization (EC-D), *p* = 1; EC-D vs. EC-hyperpolarization (EC-H), *p* = 1; control vs. EC-H, *p* = 0.176; Day2: control vs. EC-D, *p* = 1; EC-D vs. EC-H, *p* = 1; control vs. EC-H, *p* = 1; Day3: control vs. EC-D, *p* = 1; EC-D vs. EC-H, *p* = 1; control vs. EC-H, *p* = 1. (C) Summary bar graph shows average time spent on each quadrant during Probe 1 session (control, *n* = 10; EC-depolarization (EC-D), *n* = 8; EC-hyperpolarization (EC-H), *n* = 10). Repeated measures one-way ANOVA followed by Holm-corrected post-hoc comparisons measured for time spent in quadrants: control, NE vs. SW(T), *p* < 0.001, SE vs. SW(T), *p* = 0.004, NW vs. SW(T), *p* = 0.024; EC-D, NE vs. SW(T), *p* = 1, SE vs. SW(T), *p* = 1, NW vs. SW(T), *p* = 1; EC-H, NE vs. SW(T), *p* < 0.001, SE vs. SW(T), *p* < 0.001, NW vs. SW(T), *p* < 0.001. (D) Average discrimination index from Probe 1 session, calculated from the target and non-target quadrants. One-way ANOVA followed by Tukey’s pairwise comparisons, control vs. EC-D, *p* = 0.009; EC-D vs. EC-H, *p* < 0.001; control vs. EC-H, *p* = 0.256. Blue text shows the results of one-sample Student’s t-tests compared with a chance level of 0 (control, *p* < 0.001; EC-D, *p* = 0.897; EC-H, *p* < 0.001). (E) Summary bar graph shows average time spent on each quadrant during Probe 2 session (control, *n* = 10; EC-depolarization, *n* = 8; EC-hyperpolarization, *n* = 10). Repeated measures ANOVA followed by Holm-corrected post-hoc comparisons measured for time spent in quadrants; control, NE vs. SW(T), *p* = 1, SE vs. SW(T), *p* = 1, NW vs. SW(T), *p* = 1; EC-D, NE vs. SW(T), *p* = 1, SE vs. SW(T), *p* = 1, NW vs. SW(T), *p* = 1; EC-H, NE vs. SW(T), *p* = 0.001, SE vs. SW(T), *p* = 0.015, NW vs. SW(T), *p* < 0.001. (F) Average discrimination index from Probe 2 session. One-way ANOVA followed by Tukey’s pairwise comparisons. Control vs. EC-D, *p* = 0.181; EC-D vs. EC-H, *p* = 0.004; control vs. EC-H, *p* = 0.158. Blue text shows the results of one-sample Student’s t-tests in comparison to the chance level of 0 (control, *p* = 0.230; EC-D, *p* = 0.395; EC-H, *p* < 0.001). Data are represented as mean ± SEM. The reported sample sizes correspond to the number of mice. n.s. *p* > 0.05, ∗*p* < 0.05, ∗∗*p* < 0.01, and ∗∗∗*p* < 0.001.

During the first probe trial, the control and the EC-hyperpolarization groups spent significantly longer times in the target quadrant than in the other quadrants, suggesting that these mice successfully consolidated the spatial memory of the platform from the training sessions (control: F(3, 27) = 8.24 *p* < 0.001; EC-hyperpolarization: F(3, 27) = 27.546, *p* < 0.001; Figure 2C). On the contrary, the EC-depolarization group could not distinguish the target quadrant compared to other quadrants, suggesting impaired spatial memory consolidation (F(3, 21) = 0.429, *p* = 0.735). The discrimination indices showed that the EC-depolarization group had reduced ability to differentiate the target quadrant compared to the control and chemogenetic hyperpolarization groups (F(2, 25) = 11.724, *p* < 0.001; Figure 2D). The second probe trial was conducted to measure the retention of the long-term memory formed a week earlier. During the second probe trial, the control and TA-depolarization groups did not spend significantly more time in the target quadrant compared to other quadrants (control, F(3, 27) = 0.908, *p* = 0.45; EC-depolarization, F(3, 21) = 0.941, *p* = 0.439; Figure 2E). Interestingly, the EC-hyperpolarization group spent significantly more time in the target quadrant, suggesting improved consolidation of the memory from the training session (F(3, 27) = 9.483, *p* < 0.001). The discrimination index from the EC-hyperpolarization group was significantly higher than the EC-depolarization groups (F(2, 25) = 7.505, *p* = 0.003; Figure 2F). These results suggest that chemogenetic depolarization of EC during sleep following training sessions reduces consolidation of spatial memory acquired during training, consistent with its suppressive effect on TA pathway delta oscillations. On the other hand, chemogenetic hyperpolarization had no significant effect on performance during the first probe trial but significantly improved performance during the second probe trial, suggesting that the enhanced consolidation allows the memory to last longer.

### Chemogenetic hyperpolarization of TA pathway neurons enhances memory consolidation efficiency in cognitively challenging activity

Next, we investigated whether chemogenetic hyperpolarization improves rodents’ performance on a cognitively challenging task. The Y-maze space reference memory task is a well-established hippocampus-dependent memory task.^30^^,^^31^^,^^32^ The Y-maze space reference task begins with the training phase, during which the mouse is introduced to the start arm and allowed to freely explore the Y-maze with one arm blocked (Figure 3A). After a set retention delay (e.g., 2 to 24 h) following the training phase, the testing phase occurs. During the testing phase, the mouse has access to all three arms and exhibits a preference for the novel arm due to its innate novelty-seeking. The preference for the novel arm exploration is considered a measure of long-term spatial recognition memory from the training session (Figure 3A).

**Figure 3:**
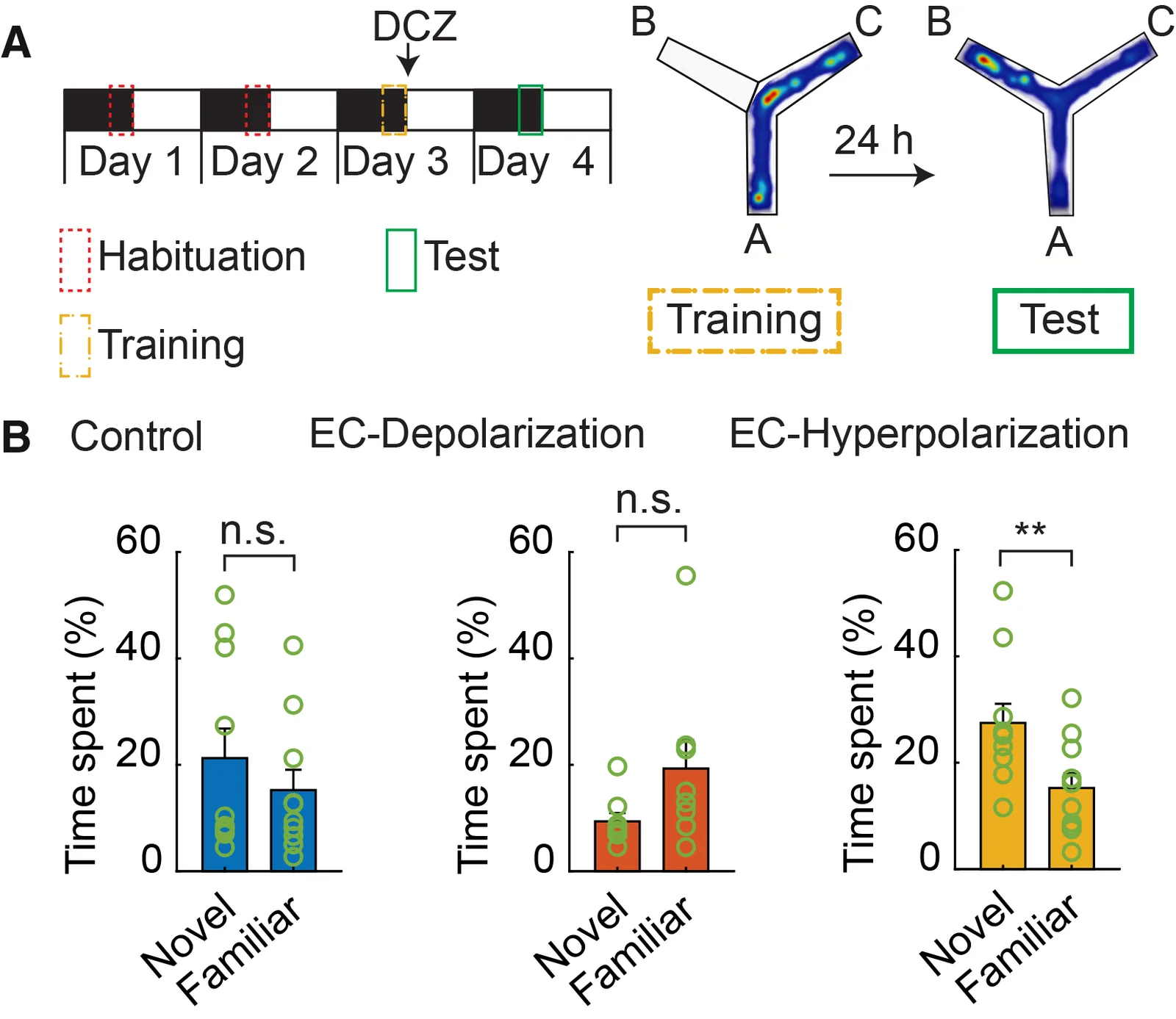
Chemogenetic hyperpolarization of EC during sleep following the training phase facilitates memory consolidation efficiency in the Y-maze space reference task with a 24 h delay (A) Schematic showing the timeline of the Y-maze space reference task and representative heatmap showing the mouse’s cumulative time spent on the maze during the training and testing phase. (B) Summary of the cumulative time spent on the familiar and novel arms (control, *n* = 10; EC-depolarization (EC-D), *n* = 8; EC-hyperpolarization (EC-H), *n* = 10). Data are represented as mean ± SEM. The reported sample sizes correspond to the number of mice. Paired Student’s *t* test, n.s. *p* > 0.05 and ∗∗*p* < 0.01.

One advantage of the Y-maze space reference task is that the difficulty of the task can be controlled by adjusting the retention delay duration. Previous studies have shown that mice can perform well with the Y-maze space reference task with a 2–4 h of retention delay.^32^ We used this challenging version of the space reference task with a 24-h retention delay to investigate whether chemogenetic hyperpolarization would improve performance. AAV carrying hM3Dq, hM4Di, or a fluorophore was injected into layer III of EC in adult male mice at least four weeks before the space reference task, and DCZ (0.1 mg/kg) was injected i.p. immediately following the training phase but before sleep (Figure 3A). The control and EC-depolarization mice did not differ significantly in the time spent in the novel versus familiar arms (control, t (9) = 0.891, *p* = 0.396; EC-depolarization, t (7) = −1.611, *p* = 0.151 Student’s paired *t* test; Figure 3B). Interestingly, mice with hM4Di activation in EC spent significantly more time on the novel arm than on the familiar arm during the testing phase (t (9) = 3.311, *p* = 0.009, Student’s paired *t* test). Taken together, chemogenetic hyperpolarization of the TA pathway facilitates the consolidation of new spatial information and enhances performance on cognitively challenging tasks, which the control mice do not perform without chemogenetic receptor activation.

### Chemogenetic modulation has no significant effect on spatial working memory

The next question was whether the effect of chemogenetic modulation is specific to the consolidation of long-term memory or also modulates short-term, working memory. To investigate the effect of chemogenetic modulation of EC on working memory, we subjected the mice to the Y-maze spontaneous alternation task. This task is based on the innate curiosity of the mice to explore previously unvisited areas.^27^^,^^33^^,^^34^ During the task, mice are allowed to freely explore all three arms of the Y-maze for a duration of 8 min without any retention delay, and the alternation rate during the task is used as an indicator of spatial working memory. Mice received intracranial injection surgery with AAV carrying hM3Dq, hM4Di, or a fluorophore in the ECIII at least two weeks before the task. For chemogenetic modulation, mice received an i.p. injection of DCZ (0.1 mg/kg) 30 min before the Y maze spontaneous alternation task (Figure 4A). The chemogenetic depolarization and hyperpolarization groups showed alternation (F(2, 26) = 0.06, *p* = 0.942; Figure 4B), direct (F(2, 26) = 1.407, *p* = 0.263; Figure 4C), and indirect visit rates (F(2, 26) = 0.49, *p* = 0.618; Figure 4D) that are not significantly different from those of the control group. These results suggest that chemogenetic modulation of the TA pathway is specific to the consolidation of long-term memory without affecting spatial working memory.

**Figure 4:**
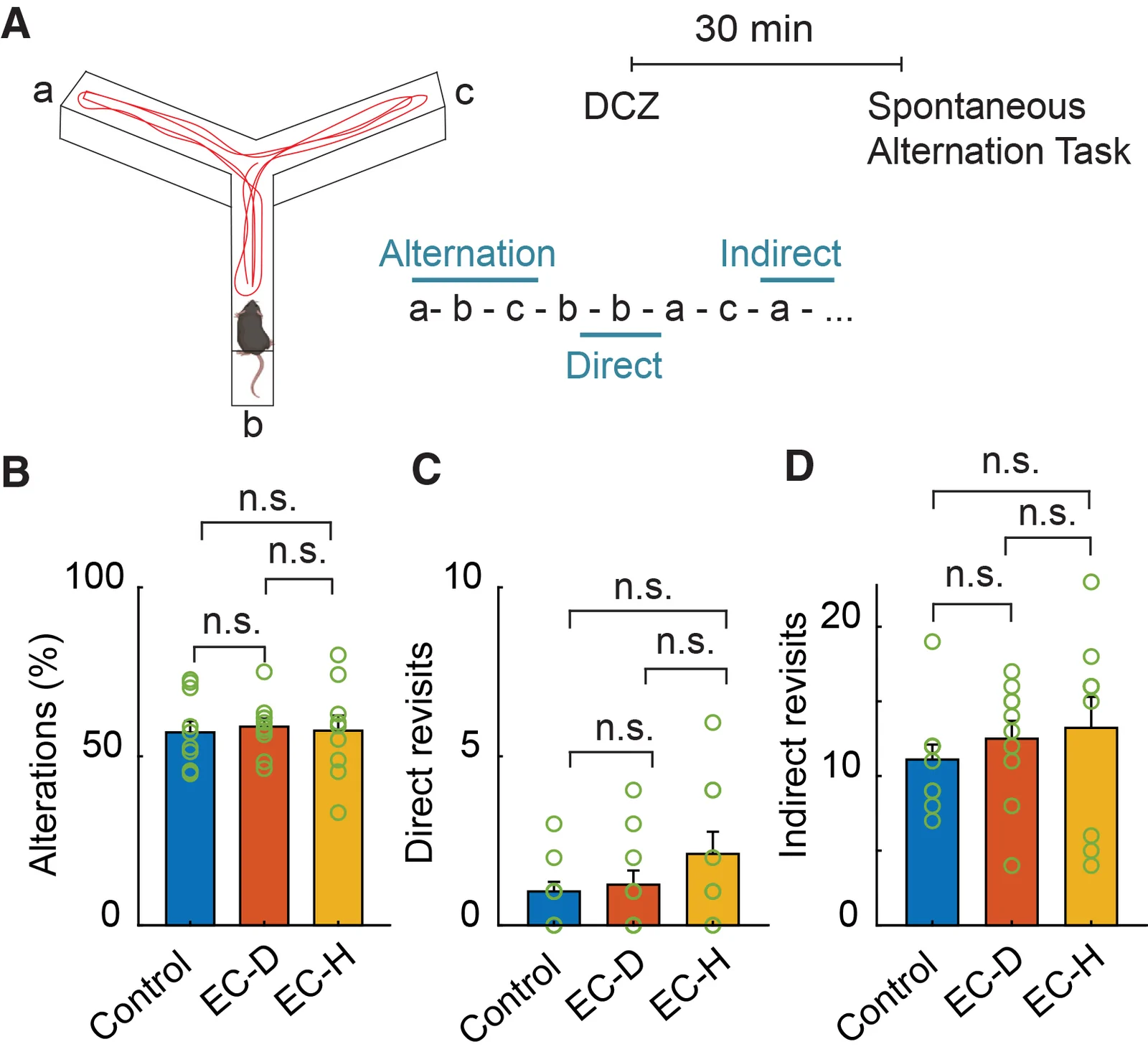
Chemogenetic manipulation of EC had no significant effect on working memory (A) Schematic showing the timeline of the DCZ injection and Y-maze spontaneous alternation task. (B) Summary graph shows the mean spontaneous alternation rate (%) from the control (*n* = 10), EC-depolarization (EC-D) (*n* = 10), and EC-hyperpolarization (EC-H) (*n* = 9) groups. One-way ANOVA followed by Tukey’s pairwise comparisons. Control vs. EC-D, *p* = 0.939; EC-D vs. EC-H, *p* = 0.971; control vs. EC-H, *p* = 0.995. (C) Summary graph shows the average number of direct visits. One-way ANOVA followed by Tukey’s pairwise comparisons. Control vs. EC-D, *p* = 0.27, EC-D vs. EC-H, *p* = 0.408, control vs. EC-H, *p* = 0.954. (D) Summary graph shows the average number of indirect visits. One-way ANOVA followed by Tukey’s pairwise comparisons. Control vs. EC-D, *p* = 0.791; EC-D vs. EC-H, *p* = 0.942; control vs. EC-H, *p* = 0.604. Data are represented as mean ± SEM. The reported sample sizes correspond to the number of mice. n.s. *p* > 0.05.

## Discussion

We provide evidence that chemogenetic hyperpolarization, typically considered an inhibitory modulation, promotes delta oscillations in the TA pathway of EC and enhances memory consolidation. Our previous work demonstrated that delta oscillations in the EC drive the consolidation of newly encoded memory.^15^ While hyperpolarization reduces neuronal spiking, it paradoxically increases EC delta activity through HCN channel activation; this increased oscillatory activity appears to be the key determinant of the behavioral output. This corroborates the functional role of neural oscillations in memory consolidation, which outweighs the spiking activity when the two have opposing effects. Likewise, chemogenetic depolarization, while increasing spiking activity, suppresses memory consolidation, likely due to reduced delta oscillations in the TA pathway.

Neural oscillations measured with calcium indicators are distinct from those measured with *in vivo* electrophysiology. In the field of neuroscience, neural oscillations in deep brain regions are typically measured using local field potential recordings, which primarily reflect synaptic input-driven changes in membrane potential across hundreds to thousands of neurons.^35^ In this study, we used genetically expressed calcium indicators to record population neural activity from glutamatergic neurons in the EC. Calcium levels are commonly used as a proxy for neural activity, as neuronal action potential firing increases calcium levels via voltage-gated calcium channels.^36^^,^^37^ Thus, neural oscillations recorded using calcium indicators mainly reflect neuronal spiking activity.^15^ This also aligns with the fact that delta oscillations of the EC could be recorded using optical probes targeting axon terminals in this study; excitatory and inhibitory postsynaptic potentials do not survive at axon terminals, whereas action potentials are actively generated due to high levels of voltage-gated channels.

The effect of chemogenetic manipulation on neural oscillations was specific to delta oscillations, without altering theta oscillations. Thus, the effect of chemogenetic receptor activation cannot be attributed to a global increase or decrease in neural activity. Instead, this selectivity reflects the unique biophysical mechanism of delta oscillations in the EC, which relies on HCN-mediated resonance that is preferentially engaged during sleep states associated with memory consolidation. Consistent with this interpretation, chemogenetic manipulation during active learning in the wake state did not impair or enhance the working memory task performance.

Given that we employed engineered G protein-coupled receptor signaling to modulate membrane potential, is it possible that the effect on delta oscillations is due to modulation of second messengers? There are four subtypes of HCN channels (HCN1-4), which display distinct gating properties. In addition to voltage-dependency, the gating of HCN channels is dependent on the second messenger cyclic adenosine monophosphate (cAMP). The binding of cAMP to the HCN channel increases the likelihood of channel opening at more depolarized potentials. This cAMP-induced shift in the gating voltage is largest with subtypes 2 and 4 (i.e., 10–20 mV), with minimal modulation on HCN1 (i.e., 1–2 mV).^16^^,^^38^^,^^39^ In the EC, HCN1 is the most predominantly expressed form.^40^^,^^41^^,^^42^ In our study, we showed that hM4Di, which causes a reduction in cAMP, causes an increase in the power of delta oscillations. Thus, the increase in delta is unlikely due to a reduction in cAMP, which would have the opposite effect on delta. Likewise, activation of hM3Dq, which causes membrane depolarization via a cAMP-independent pathway, caused a reduction in delta oscillations, further supporting that the membrane potential, not cAMP, is the primary driver of the effect.

In summary, our study demonstrated that hM4Di activation increases delta oscillations and promotes the consolidation of hippocampus-dependent memory. These results suggest that while membrane potential is typically considered an indicator of neuronal excitability and activation of the circuit, neural synchrony, manifesting as oscillations, serves as a critical neural code in the EC that determines successful memory consolidation.

### Limitations of the study

In this study, we could not measure the effect of chemogenetic modulation specifically on encoding, as compared to its effect on consolidation. This is due to the fact that the encoding session is short, and the chemogenetic receptor activated during the session will remain active during the consolidation phase. However, performance in the Y-maze spontaneous alternation task demonstrated that chemogenetic modulation of the EC neurons did not alter short-term working memory, supporting the conclusion that its facilitatory effect is selective for long-term memory consolidation. Another limitation of this study is that only male mice were used in this study, and the findings reported here may not generalize to females. To determine whether the effect of chemogenetic modulation of entorhinal cortical neurons on delta oscillations and memory consolidation extends across sexes, future studies involving females are needed.

## Resource availability

### Lead contact

Further information and requests for resources and reagents should be directed to and will be fulfilled by the lead contact, Juhee Haam (jhaam1@lsu.edu).

### Materials availability

This study did not generate new unique reagents.

### Data and code availability

• All data reported in this paper will be shared by the lead contact upon request.
• All original code has been deposited on GitHub and is publicly available at https://github.com/haam-lab/Gunin_etal_2026_TA_chemogenetics.
• All other items will be available from the lead contact upon request.

## Acknowledgments

We thank Drs. Negin Martin and Bo He at the NIEHS Viral Vector Core for preparing the virus. We thank the LSU Advanced Microscopy and Analytical Core for assistance with imaging and all members of the Haam lab for help with the daily operation of the laboratory. This work was supported by the LSU College of Science and Department of Biological Sciences Startup funds (J.H.), the Board of Regents Research Competitiveness Support Program, LEQSF(2022-25)-RD-A-08 (J.H.), and NIH R01 (1R01MH140001). (J.H.).

## Author contributions

J.H. conceived the studies. S.G. and J.H. designed the studies; S.G., J.V., and J.H. conducted experiments; S.G., J.V., and J.H. analyzed data; J.H. supervised the studies; and S.G. and J.H. wrote the manuscripts.

## Declaration of interests

The authors declare no competing interests.

## STAR★Methods

### Key resources table

**Table:** 

REAGENT or RESOURCE	SOURCE	IDENTIFIER
**Antibodies**		

Rabbit monoclonal anti-GAD65 + GAD67 (clone EPR19366), 1:500	Abcam	Cat# ab183999; RRID: AB_3662875
Guinea pig polyclonal anti-NeuN, 1:250	Synaptic Systems	Cat# 266 004; RRID: AB_2619988
Goat anti-rabbit IgG (H + L) Alexa Fluor® 647, 1:500	Thermo Fisher Scientific	Cat# A-21245; RRID: AB_2535813
Goat anti-guinea pig IgG (H + L) Alexa Fluor® 488, 1:500	Abcam	Cat# ab150185; RRID: AB_2736871

**Bacterial and virus strains**		

AAV9-CaMKIIα-jGCaMP8f-WPRE	NIEHS Viral Vector Core	Addgene: 176750
AAV9-CaMKIIα-GCaMP6f	NIEHS Viral Vector Core	N/A
AAV9-CaMKIIα-tdTomato-WPRE	NIEHS Viral Vector Core	N/A
AAV9-CaMKIIα-hM4D(Gi)-mCherry	NIEHS Viral Vector Core	Addgene: 50477
AAV9-CaMKIIα-hM3D(Gq)-mCherry	NIEHS Viral Vector Core	Addgene: 50476
AAV9-hSyn-DIO-mCherry	NIEHS Viral Vector Core	Addgene: 50459

**Chemicals, peptides, and recombinant proteins**		

Deschloroclozapine (DCZ) Dihydrochloride	NIMH Chemical Synthesis and Drug Supply Program	Cat# D-925; CAS 1977–07-7
Isoflurane	Vetone	NDC 13985–528-60
Ketamine hydrochloride	Vetone	NDC 13985–584-10
Xylazine (Rompun)	Dechra	NDC 17033–099-05
Carprofen	Vetone	NDC 13985–748-20
Pentobarbital sodium (Euthaphen)	Dechra	NDC 17033–088-10
Paraformaldehyde	Electron Microscopy Sciences	Cat# 15714
Normal goat serum	Jackson Immuno Research	Cat# 005–000-121
Fluoro-Gel with DABCO	Electron Microscopy Sciences	Cat# 17985–02
*N-*methyl-D-glucamine (NMDG)	Thermo Scientific	Cat# 126845000
HEPES	AmBeed	Cat# A318293
Potassium gluconate	Thermo Scientific	Cat# B25135.30
Mg-ATP	Sigma Aldrich	Cat# A9187
Na_2_GTP	Sigma Aldrich	Cat# G8877
EGTA	Thermo Scientific	Cat# A16086.09
Sorbitol	Tokyo Chemical Industry	Cat# S0065
Sucrose	Fisher Scientific Chemical Division	Cat# BP220-212

**Experimental models: Organisms/strains**		

C57BL/6J	The Jackson Laboratory	JAX: 000664

**Software and algorithms**		

MATLAB 2022, 2023, 2024,2025	MathWorks	RRID: SCR_001622
EthoVision XT16 and 17	Noldus	RRID: SCR_000441
JASP (v0.16.3 and v0.96)	JASP Team	RRID: SCR_015823
Clampex 11.3 (pCLAMP 11)	Molecular Devices	RRID: SCR_011323
Fiji (ImageJ 1.54f)	https://imagej.net/software/fiji/	RRID: SCR_002285
Video-based sleep detection algorithm	https://github.com/haam-lab/Haam_etal_TA_consolidation	N/A
Photometry data processing algorithm	https://github.com/haam-lab/Haam_etal_TA_consolidation	N/A
Ex vivo electrophysiology data processing algorithm	https://github.com/haam-lab/Gunin_etal_2026_TA_chemogenetics	N/A

### Experimental model and study participant details

We obtained wild-type C57B6/6J mice from the Jackson Laboratory (strain #000664) and bred them in-house in the Louisiana State University animal facility. In this study, we used 3 to 8-month-old male mice. Mice were group-housed until surgery and single-housed thereafter to protect the implanted optical probes for the photometry experiments. All mice were kept group-housed before and after the virus injection surgery for the behavioral experiments. All experiments were approved by and conducted in accordance with the Louisiana State University Institutional Animal Care and Use Committee (protocol number IACUCAM-24–032). All mice were housed on a 12h light/12h dark cycle (lights on at 12 p.m.) at 20°C–23°C and 45–65% relative humidity, with food and water available *ad libitum*. The mice were inspected weekly by veterinarians, and pathogen-free conditions were maintained across all colonies.

### Method details

#### Intracranial surgery for virus injection

Before viral injection surgery, mice were anesthetized in an induction chamber with 4% isoflurane, followed by intraperitoneal injection of ketamine/xylazine (100 mg/kg ketamine, 7 mg/kg xylazine). After stabilization of respiration, mice were secured in a stereotaxic apparatus (Stoelting). A 5 μL Hamilton syringe (30 G) was used to inject 500 nL of virus per site at a rate of 100 nL/min using a syringe pump. To avoid backflow of the viral solution after infusion, the syringe remained in place undisturbed for 5 min before being withdrawn from the injection site. To investigate the effect of chemogenetic hyperpolarization on the neural activity of the TA pathway, mice received bilateral injection of a virus cocktail of AAV9-CaMKIIα-GCaMP6f, AAV9-CaMKIIα-tdTomato-WPRE, and AAV9-CaMKIIα-hM4Di-mCherry into layer III of the EC (AP-0.5; ML ± 3.65; DV, 4.30; the lambda as a reference). Similarly, to examine the effect of chemogenetic depolarization on the TA neural activity, a virus cocktail of AAV9-CaMKIIα-GCaMP6f, AAV9-CaMKIIα-tdTomato-WPRE, and AAV9- CaMKIIα-hM3Dq-mCherry was injected at the same coordinates. For behavioral studies examining the effect of chemogenetic modulation, mice received bilateral injections of AAV9-CaMKIIα-hM4Di-mCherry (hyperpolarization) or AAV9-CaMKIIα-hM3Dq-mCherry (depolarization) in EC layer III. The control group received either AAV9-CaMKIIα-tdTomato AAV9-hSyn-DIO-mCherry. A recovery period of at least 3 weeks was allowed for the mice to express the AAV9 viral construct before behavioral testing. For *ex vivo* electrophysiological recordings, mice were bilaterally injected with AAV9-CaMKIIα-hM3Dq-mCherry in layer III of the EC (AP-0.5; ML ± 3.65; DV, 4.30; the lambda as a reference).

#### *In vivo* photometry

To measure signals from fluorophores *in vivo*, we used a custom, spectrometer-based photometry setup.^15^^,^^26^ In short, a 488 nm laser with a tip power output of 70–75 μW was used to excite GCaMP6f and tdTomato via optical probes implanted in mice. The emitted signals were routed via a dichroic mirror to a spectrometer (Ocean Optics, QE-Pro). To eliminate ambient light detection, background subtraction was conducted with the room lights off before connecting the patch cable to an optical implant on a mouse.

Bilateral optical probe implantations were conducted after a 3-week recovery period following viral injections. Anesthesia was induced as described in *Intracranial Surgery for Virus Injection* and maintained at 0.4% Isoflurane supplied at a rate of 0.8 L/min through a nose cone in the stereotaxic setup. To record from TA pathway terminals, optical probes (200 μm core, 230 μm cladding, NA 0.48) were implanted in the CA1 area (AP -2.35, ML ±2.25, DV -1.5) such that the probes were placed 500 μm dorsal to the region of interest. During optical probe implantation surgery, an optical probe was attached to a stereotaxic arm and lowered slowly to the target region while recording the signal online. The presence of characteristic anesthesia-state oscillations confirmed the recordings to be from TA pathway neurons. After being placed at the target site, the optical probes were anchored to the skull with dental cement. Mice were allowed to recover by placing them on a heating pad until ambulatory. *In vivo* recordings were carried out in these mice after at least 2 weeks of recovery.

*In vivo* photometry recordings were conducted in the home cage during the early phase of the light cycle. To acquire the video and photometry data simultaneously, we used a custom-made Arduino setup that sends 25Hz TTL signals to the recording devices.^15^ The first day of baseline recording without any treatment was followed by the second day of recording, which occurred 30 min after an intraperitoneal (i.p.) injection of saline (0.1 cc/10 g body weight), the third day of recording, 30 min after i.p. injection of DCZ (0.1 mg/kg), and the last day of recording without any injection (washout).

#### Behavioral studies

All behavioral studies were done 2–3 h before the beginning of the light cycle. To familiarize mice with the experimental room, they were brought in at least 30 min before the start of each behavioral study. Before any behavioral testing, mice were handled for at least 4 days to ensure they were not anxious during testing. During the handling period, mice were scuffed and mock-injected with saline (0.1cc/10 g body weight) i.p.

The Y-Maze (SD Instruments) consisted of three gray closed arms measuring 35 cm L × 5 cm W × 20 cm H, joined at 120°. The Y-maze was placed in a curtained area with distinct extramaze spatial cues. The Y-maze space reference memory tasks consist of training and testing phases. During the training phase, with one designated arm blocked, the mouse was placed in the start arm and allowed to freely explore the remaining two arms of the Y maze. After 10 min of exploration, each mouse was placed back in its home cage and was injected with DCZ i.p (0.1 mg/kg). After a retention delay of 24 h, the testing phase occurred, during which the mice were allowed to explore all three arms for 10 min. The blocked arm was counterbalanced across all groups. The Y-maze was cleaned with 70% ethanol before and after each animal and wiped dry. The time spent in the novel and familiar arms were analyzed using EthoVision XT.

The Y-maze spontaneous alternation was conducted by placing the mouse in the center of the Y-maze and allowing the mouse to freely explore the entire maze for 8 min. Mice’s arm visits were automatically detected, and alternation, direct, and indirect visit rates were acquired using EthoVision XT with the exit threshold of 4 cm.

The Morris water maze task consists of the training phase and two probe trials. The water maze (SD Instruments) used in this study was a circular pool with a diameter of 150 cm and a height of 81 cm, filled with 21 ± 1°C water. The water maze was placed in an enclosed area with curtains and extramaze cues. One day before the training phase, a habituation session was conducted to allow mice to become familiar with the spatial cues and to become aware of the platform. During the habituation session, a visible platform was positioned in the center of the water maze, and each mouse was placed on the platform for 60 s. During the training phase of three days, a transparent glass platform was submerged 1–1.5 cm below the water’s surface. Each training day, mice completed three blocks, with each block containing four trials. Mice were placed in a random quadrant for each trial, and mice had to find the platform to escape the water within 60 s. Mice that fail to find the platform within 60 s were guided to the hidden platform and allowed to sit on the platform for 15 s before being dried off with a microfiber cloth. After the end of the second and third training days, mice were administered DCZ (0.1 mg/kg) i.p. The first and second probe trials occurred one day and eight days after the last training day, respectively. During the probe trials, mice were placed in the opposite quadrant where the platform had been during the training phase and were allowed to swim freely for a period of 60 s without any platform. The location of mice was tracked using EthoVision XT, and latency to the platform, time spent in each quadrant, total distance moved, and velocity were analyzed. The discrimination index was calculated by the following:

$$\frac{T_{Target}-T_{NonTarget}}{T_{Target}+T_{NonTarget}}\times 100$$

where T_Target_ is the time spent on the target quadrant, whereas T_NonTarget_ is the average of time spent on three non-target quadrants. The daily learning rate for the training days was calculated as:

$$\frac{T_{Block1}-T_{Block3}}{T_{Block1}}$$

where T_Block1_ is the average escape latency in block 1, and T_Block3_ is the average escape latency in block 3. Similarly, the Day 3 learning rate between block 1 and block 2 was calculated as:

$$D3\;LR⎼B12=\frac{T_{Block1}-T_{Block2}}{T_{Block1}}$$

where T_Block1_ is the average escape latency in block 1, and T_Block2_ is the average escape latency in block 2. Day 3 learning rate between block 2 and block 3 was calculated as:

$$D3\;LR⎼B23=\frac{T_{Block2}-T_{Block3}}{T_{Block2}}$$

where T_Block2_ is the average escape latency in block 2, and T_Block3_ is the average escape latency in block 3.

#### *Ex vivo* electrophysiology

Mice were euthanized 4 weeks post-virus injection, between 10 and 14 weeks old. Prior to slice preparation, mice were anesthetized by the open-drop method, and transcardial perfusion was performed once the pedal-withdrawal reflex was abolished. Ice-cold cutting ACSF (92 mM *N-*methyl-*D*-glucamine (NMDG), 2.5 mM KCl, 1.25 mM NaH_2_PO_4_, 30 mM NaHCO_3_, 20 mM HEPES, 2 mM thiourea, 25 mM glucose, 0.5 mM CaCl_2_·2H_2_O, 10 mM MgSO_4_·7H_2_O, 5 mM Na-ascorbate, 3 mM Na-pyruvate; pH 7.3) was used for perfusion. The mouse brain was rapidly extracted and placed in a carbogen-saturated (95% O_2_/5% CO_2_) ice-cold cutting ACSF. To record from EC, horizontal sections of 350 μm were cut using a Compresstome (VF-210-0Z, Precisionary Instruments). Each section was transferred to a Petri dish containing well-oxygenated holding ACSF (92 mM NaCl, 2.5 mM KCl, 1.25 mM NaH_2_PO_4_, 30 mM NaHCO_3_, 20 mM HEPES, 2 mM Thiourea, 25 mM Glucose, 2 mM CaCl_2_·2H_2_O, 2 mM MgSO_4_·7H_2_O; pH 7.3) and cut in the midline to produce hemi-slices. The hemislices were carefully transferred to a recovery chamber containing holding ACSF at 25° C and were bubbled with carbogen. The slices were kept in the holding ACSF for 45 min before being transferred to a recording chamber, which was perfused with oxygenated recording ACSF (126 mM NaCl, 3 mM KCl, 1.2 mM NaH_2_PO_4_, 25 mM NaHCO_3_, 11 mM Glucose, 2 mM CaCl_2_·2H_2_O, 1.6 mM MgSO_4_·7H_2_O; pH 7.3). All patch-clamp recordings were performed at 25° C.

The neurons expressing AAV9-CaMKIIα-hM3Dq-mCherry in EC were identified by brief epifluorescence illumination using a Texas Red filter cube U-N49008 (EXET560/40*X*; BST585LP; EMET630/75m). Neurons that did not have any fluorescent signal were considered negative for expressing AAV9-CaMKIIα-hM3Dq-mCherry. After identifying mCherry expression, cells were patched by visualizing them through an IR-DIC 40*x* lens on a BX51WI microscope (Olympus). Whole-cell patch-clamp recordings were performed using borosilicate glass (OD 1.70 mm, ID 1.3 mm; KG-7740; King Precision Glass). The pipettes (tip resistance of 4–6 MΩ) were made with a horizontal micropipette puller (P-1000; Sutter Instruments). The patch pipette was filled with the internal solution (130 mM K-gluconate, 2 mM MgCl_2_·6H_2_O, 2 mM Mg-ATP, 0.3 mM Na_2_GTP, 10 mM HEPES, 1 mM EGTA, 10 mM glucose, 40 mM sorbitol; pH 7.2). The pipette was attached to the head-stage (CV 7B, Axon Instruments). The recordings were performed using Clampex 11.3 with a Multiclamp 700B amplifier (Molecular Devices) and Digidata 1550 B (Molecular Devices).

Deschloroclozapine Dihydrochloride (DCZ) (NIMH, D-925) was puff-applied at a concentration of 500 nM and a pressure of 5–10 psi using a glass pipette (tip resistance of 3–4 MΩ) attached to a Pneumatic PicoPump (World Precision Instruments, PV830). The puff pipette was carefully lowered using a micromanipulator and placed 5–10 μm above the target cell prior to patching. Once the cell was patched, the resting membrane potential (RMP) was recorded in the current-clamp mode. The duration of puff application was 2 s, and the onset of puff application was triggered through a Clampex protocol. The brain slice was continuously perfused with recording ACSF at a flow rate of 0.2 mL/min.

Custom MATLAB code was used to analyze patch-clamp data. Delta RMP was calculated by subtracting the baseline value from the RMP value. The baseline value was calculated as the mean RMP over the 5 s immediately prior to the onset of the puff application. To perform statistical tests on the effect of DCZ, the mean RMP of the 1-s window, which starts 1 s after the end of DCZ puff application, was compared to the mean RMP of the 1-s window immediately prior to the puff application of DCZ.

#### Histology

After the behavioral testing and *in vivo* fiber photometry recordings, mice were injected with pentobarbital (100 mg/kg) and transcardially perfused with 4% paraformaldehyde (PFA) in 0.1 M PBS. The brain was post-fixed in 4% PFA in 0.1 M PBS overnight and sectioned into 50–100 μm-thick horizontal slices using a vibrating microtome. Brain sections were imaged using a Leica SP8 or DM6B microscope to verify viral expression and optical probe placement.

#### Immunohistochemistry

Mice injected with AAV9-CaMKIIα-hM3Dq-mCherry and AAV9-CaMKIIα-hM4Di-mCherry were anesthetized by injecting pentobarbital (100 mg/kg) intraperitoneally. After loss of the pain reflex, mice were transcardially perfused with ice-cold PBS followed by ice-cold 4% PFA. Dissected brains were fixed overnight by keeping them in 4% PFA at 4 °C for post-fixation. Brains were placed in 30% sucrose solution in PBS for at least 48 h at 4 °C until the brains sank to ensure cryoprotection. Brains were frozen with the Tissue-Freezing medium and were horizontally sectioned at 40 μm thickness using a cryostat (Leica CM1850). Sections were washed in PBS for 20 min three times with gentle shaking to remove the Tissue-Freezing Medium. Sections were incubated in blocking solution (10% NGS in PBS) for 2 h at RT. The sections were then incubated with the primary antibodies for GAD65/67 (Abcam, cat# ab183999, 1:500 dilution) and NeuN (Synaptic Systems, cat# 266 004, 1:250 dilution) in 2% NGS PBS overnight at 4 °C. After washing three times with PBS, sections were incubated with Alexa fluor-conjugated secondary antibodies (1:500 dilutions) diluted in PBS for 2 h at RT in the dark. The sections were mounted with Fluoro Gel with DABCO (Electron Microscopy Sciences) and imaged using a Leica SP8 Confocal microscope (Leica Microsystems, Wetzlar, Germany) with a 40*X* oil-immersion objective lens. Images were acquired from two sections containing EC from each animal (three mice per group). For fluorophore quantification of immunostained-positive cells, object-based colocalization analysis was performed using the ImageJ/Fiji Colocalization Object Counter plugin. Predetermined thresholds (NeuN, 30; mCherry, 40; GAD65/67, 25) were used to identify NeuN, mCherry, and GAD65/67-positive cells. Cells in layer III EC were counted, and the numbers from six sections were pooled for each of the hM3Dq and hM4Di groups.

#### Neural data analysis

*In vivo* fiber photometry data were analyzed using a ratiometric approach and the multitaper method, as described previously.^15^ Briefly, the coefficients for GCaMP6f and tdTomato were acquired via linear spectral unmixing using custom MATLAB scripts. Movement artifacts were corrected by taking the ratio of the GCaMP6f coefficient to that of tdTomato. Photometry data were normalized as a percentage of F/F_0_ before being used for frequency-domain analysis with the multitaper method (a moving window width of 10 s and a step size of 1 s).

#### Video data analysis

Sleep/wake classification was performed using machine learning of video data acquired simultaneously with fiber photometry recordings, as described previously.^15^ In short, the machine learning model was trained with manually scored electroencephalogram, electromyography, and video data. Sleep-wake classification data were generated from pixel-change-based mobility features using a bagged ensemble classifier.

### Quantification and statistical analysis

Statistical analyses were performed using MATLAB (MathWorks, 2022–2025) and JASP (JASP Team, v0.16 and v0.96). To test for normality of the samples, the Shapiro-Wilk test was used. To compare two groups, Student’s *t* test was used for data that followed a normal distribution, whereas the Mann-Whitney U test was used for data that did not follow a normal distribution. To compare three or more groups, ANOVA was used as a parametric test, and the Kruskal-Wallis test was used as a non-parametric test. For pairwise comparisons, Holm’s and Tukey’s methods were used. All data were expressed as mean ± SEM. Statistical significance was defined as *p* < 0.05.
