# Supplementary material for: Chemogenetic hyperpolarization in the entorhinal cortex during sleep facilitates delta oscillations and memory consolidation
Source: iScience. 2026 Nov 5;29(11):118120. doi: 10.1016/j.isci.2026.118120 (PMC13769090; doi:10.1016/j.isci.2026.118120)
Supplement: Supplementary file 1 [file mmc1.pdf]

iScience, Volume *29*

Supplemental information

Chemogenetic hyperpolarization in the entorhinal cortex during sleep facilitates delta oscillations and memory consolidation

Suman Gunin, Judy Vu, and Juhee Haam

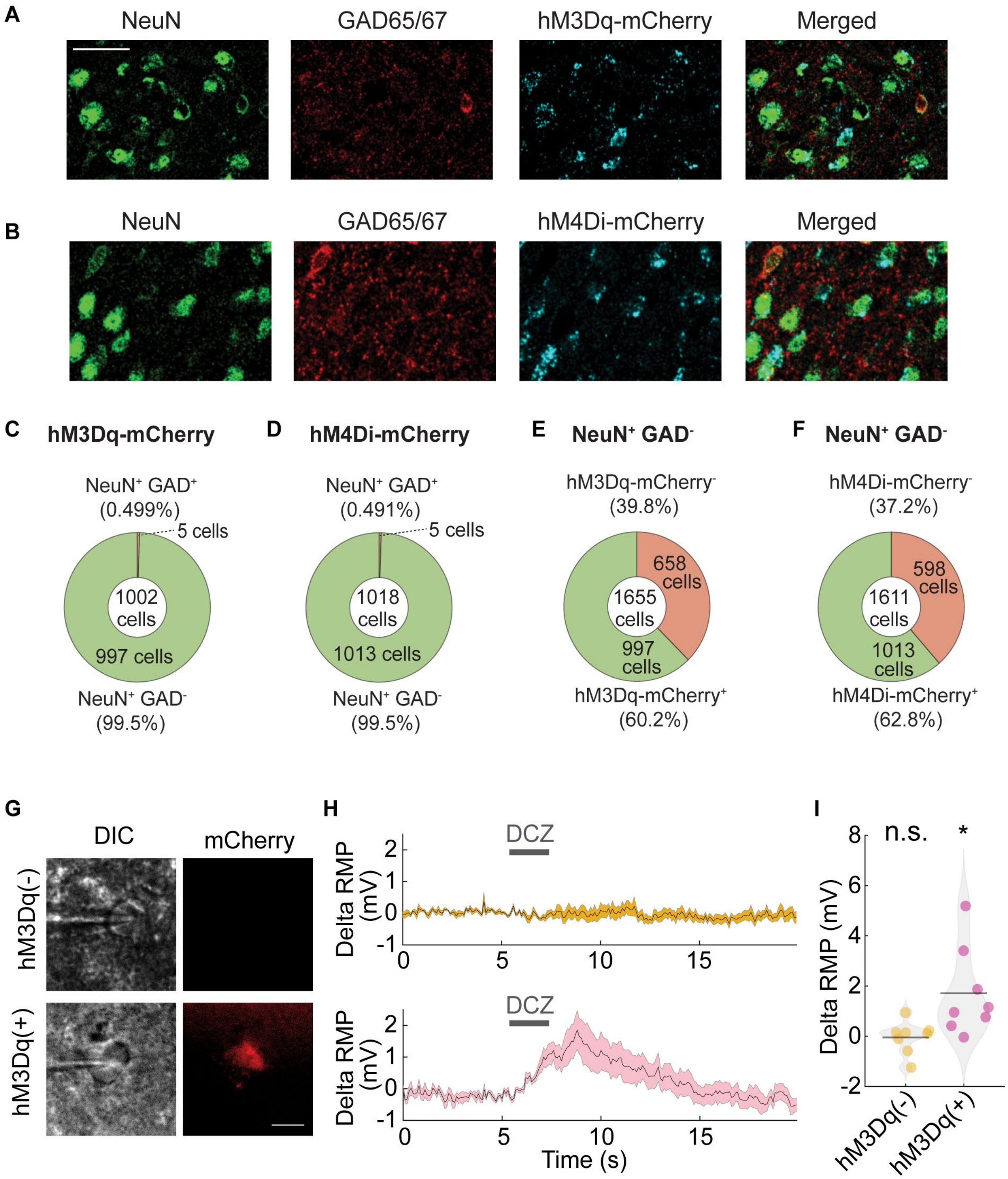

Figure S1. Expression of chemogenetic receptors and single-cell response. (A) Representative images of a brain section containing layer III entorhinal cortex (ECIII) showing the expression of AAV9-CaMKIIα-hM3Dq-mCherry (cyan), NeuN (green), and GAD65/67 (red). Scale bar, 30 μm. **(B)** Representative images of a brain section containing ECIII showing the expression of AAV9-CaMKIIα-hM4Di-mCherry (Cyan), NeuN (green), and GAD65/67 (red). The scale bar in (A) also applies to (B). **(C)** Pie chart showing the percentage of mCherry-positive hM3Dq-expressing neurons in ECIII that are GABAergic (NeuN$^+$, GAD65/67$^+$) and putative glutamatergic (NeuN$^+$, GAD65/67$^-$) (n = 1002 cells from 3 mice). **(D)** Pie chart showing the percentage of mCherry-positive hM4Di-expressing neurons in ECIII that

are GABAergic (NeuN$^+$, GAD65/67$^+$) and putative glutamatergic (NeuN$^+$, GAD65/67$^-$) (n = 1018 cells from 3 mice). **(E)** Pie graph showing the proportion of putative glutamatergic neurons (NeuN$^+$, GAD65/67$^-$) that were hM3Dq-mCherry-positive and hM3Dq-mCherry-negative (n = 1655 cells from 3 mice). **(F)** Pie graph showing the proportion of putative glutamatergic neurons (NeuN$^+$, GAD65/67$^-$) that were hM4Di-mCherry-positive and hM4Di-mCherry-negative (n = 1611 cells from 3 mice). **(G)** Representative images showing hM3Dq-mCherry-negative and hM3Dq-mCherry-positive recorded cells visualized with a 40x objective lens. Scale bar, 10 μm. **(H)** Top, time-course graph showing the average delta resting membrane potential (RMP) in mCherry-negative neurons in response to puff application of DCZ (500 nM) (n = 8 cells from 3 mice). Bottom, time-course graph showing the average delta RMP in mCherry-positive neurons in response to a puff application of DCZ (500 nM) (n = 8 cells from 3 mice). **(I)** Summary bar graph showing the average delta RMP from a 1-second post-DCZ time window, which starts 1 second after the end of DCZ application. Student's paired t-test, pre vs. post-DCZ in mCherry-negative cells, $p = 0.839$; pre vs. post-DCZ in mCherry-positive cells, $p = 0.028$. Data are represented as mean ± SEM. n.s. $p > 0.05$; *, $p < 0.05$

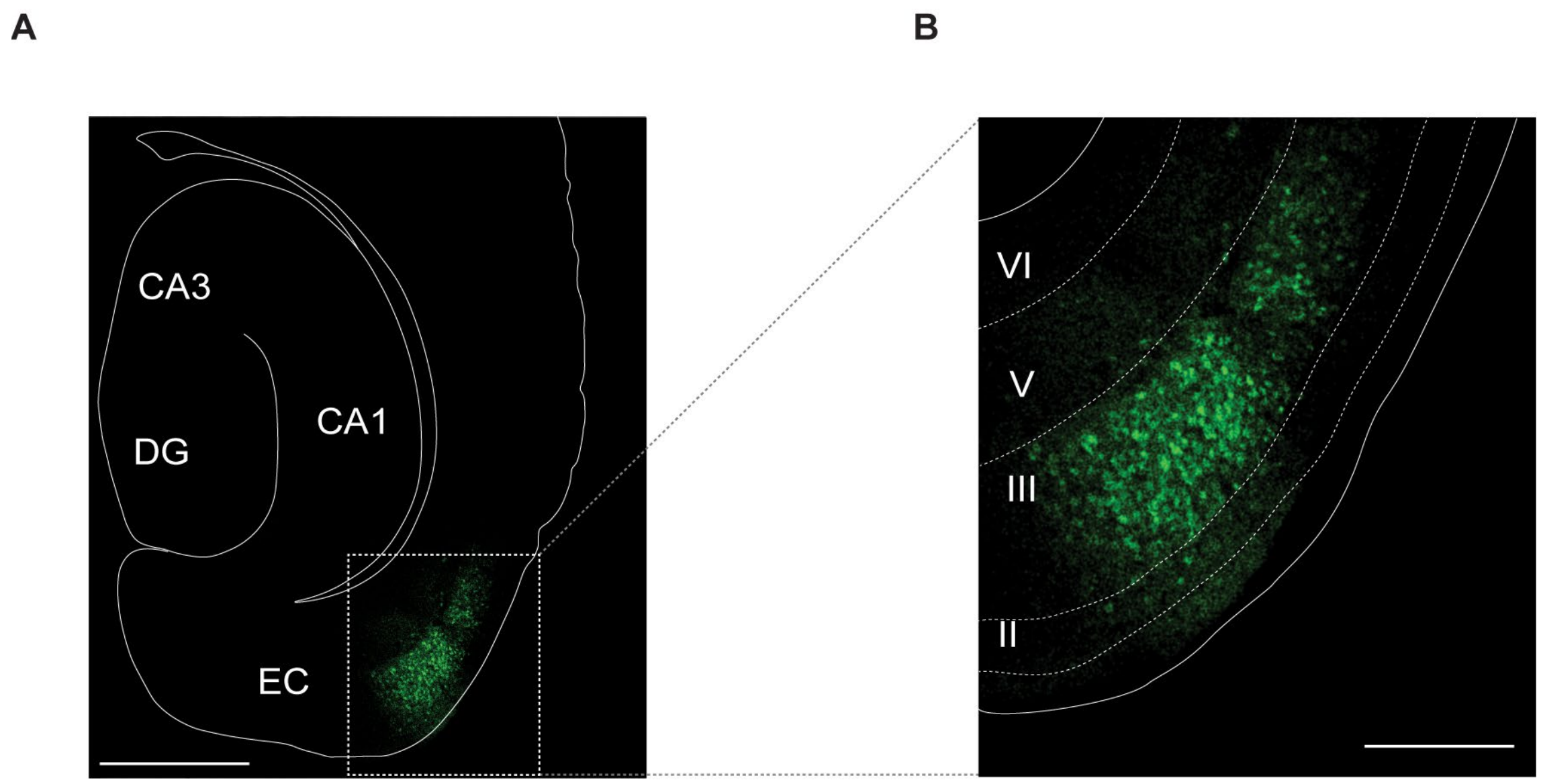

Figure S2. Representative images showing viral expression in the TA pathway neurons.
(A) A representative horizontal brain section from a mouse injected with AAV-CaMKIIα-GCaMP6f, showing the labeling in the caudomedial part of the entorhinal cortex. Scale bar, 500 μm.
(B) Magnified inset in (A), showing GCaMP6f expression in layer III entorhinal cortical neurons. Scale bar, 100 μm. Images are representative of 9 mice injected with AAV-CaMKIIα-GCaMP6f.

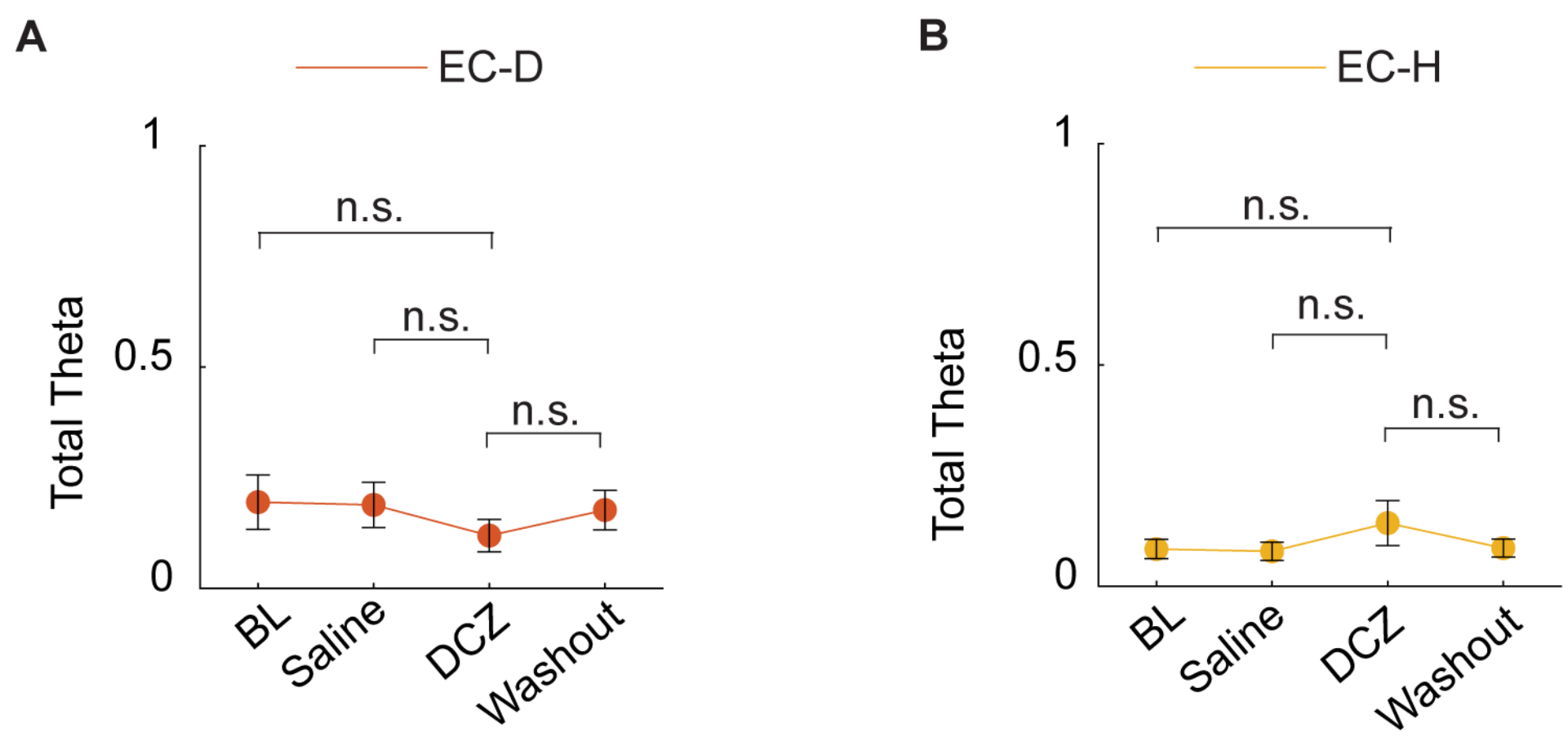

Figure S3. The effect of chemogenetic modulation on theta oscillations in the TA pathway. (A) Summary graph showing the effect of membrane depolarization of the entorhinal cortex (EC-D) via the activation of hM3Dq on theta oscillations. Average power of theta oscillations during two-hour fiber photometry recordings across four consecutive days of recordings from mice injected with AAV-CaMKIIα-hM3Dq-mCherry (n = 4). The first day of baseline recording (BL) was followed by a recording with saline injection (Saline) on the second day, with DCZ injection (DCZ) on the third day, and without any treatment (Washout) on the fourth day. Repeated measures ANOVA followed by Holm-corrected post-hoc comparisons. Baseline (BL) vs. DCZ, p = 0.066; saline vs. DCZ, p = 0.084; DCZ vs. washout, p = 0.151. **(B)** Summary graph showing the effect of membrane hyperpolarization of the TA pathway (EC-H) via the activation of hM4Di on theta. Average power of theta oscillations during two-hour fiber photometry recordings across four consecutive days of recordings from mice injected with AAV-CaMKIIα-hM4Di-mCherry (n = 5). Repeated measures ANOVA followed by Holm-corrected post-hoc comparisons. BL vs. DCZ, p = 0.21; saline vs. DCZ, p = 0.177; DCZ vs. washout, p = 0.21. Data are represented as mean ± SEM. The reported sample sizes correspond to the number of mice. n.s. p > 0.05.

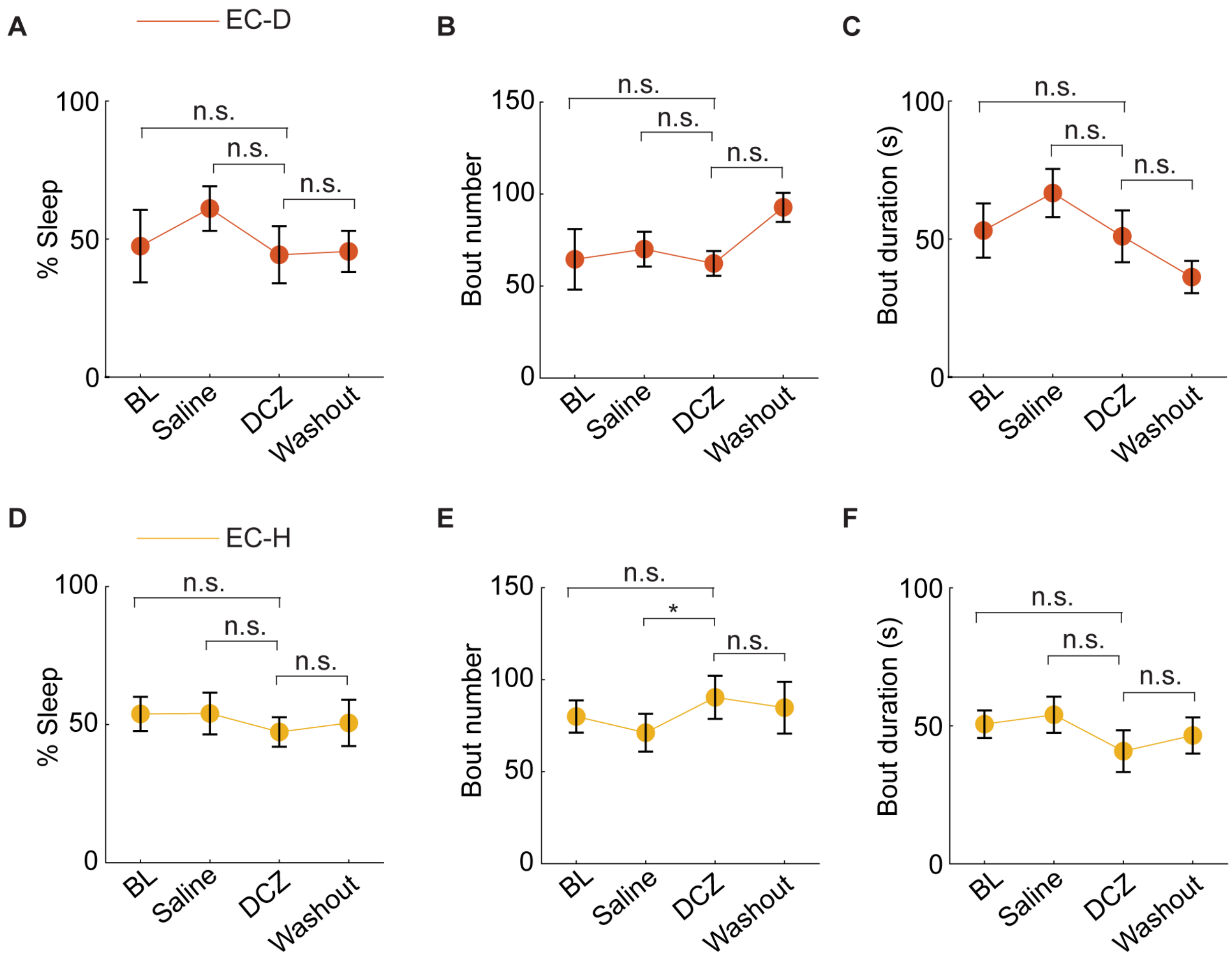

Figure S4. The effect of chemogenetic modulation on sleep architecture. (A) Summary graph showing the effect of membrane depolarization of the entorhinal cortex (EC-D) via the activation of hM3Dq on the average sleep amount (as a percentage of the total time) in mice injected with AAV-CaMKIIα-hM3Dq-mCherry (n = 4). The recordings were conducted for four consecutive days: baseline (BL, Day 1), saline injection (Saline, Day 2), DCZ injection (DCZ, Day 3), and no treatment (Washout, Day 4). Repeated measures ANOVA followed by Holm-corrected post-hoc comparisons; baseline vs. DCZ, p = 1; saline vs. DCZ, p = 0.271; DCZ vs. washout, p = 1. **(B)** Summary graph showing the effect of EC-D on the average sleep bout number. Repeated measures ANOVA followed by Holm-corrected post-hoc comparisons; baseline vs. DCZ, p = 1; saline vs. DCZ, p = 1; DCZ vs. washout, p = 0.074 (n = 4). **(C)** Summary graph showing the effect of EC-D on the average sleep bout duration in seconds. Repeated measures ANOVA followed by Holm-corrected post-hoc comparisons; baseline vs. DCZ, p =0.837, saline vs. DCZ, p = 0.605; DCZ vs. washout, p = 0.605 (n = 4). **(D)** Summary graph showing the effect of membrane hyperpolarization of the entorhinal cortex (EC-H) via the activation of hM4Di on the average sleep amount (in percentage of the total time) in mice injected with AAV-CaMKIIα-hM4Di-mCherry (n = 5, each mouse represents an independent biological replicate), Repeated measures ANOVA followed by Holm-corrected post-hoc comparisons; baseline vs. DCZ, p = 1; saline vs. DCZ, p = 1; DCZ vs. washout, p = 1. **(E)** Summary graph showing the effect of EC-H on the average sleep bout number. Repeated measures ANOVA followed by Holm-corrected post-hoc comparisons; baseline vs. DCZ, p = 0.238, saline vs. DCZ, p = 0.014, DCZ vs. washout, p = 0.569 (n = 5). **(F)** Summary graph showing the effect of EC-H on the average sleep bout duration in seconds. Repeated measures ANOVA followed by Holm-corrected post-hoc comparisons; baseline vs. DCZ, p = 0.729; saline vs. DCZ, p = 0.347; DCZ vs. washout, p = 0.1 (n = 5). Data are

represented as mean ± SEM. The reported sample sizes correspond to the number of mice. n.s. $p > 0.05$, $^*p < 0.05$.

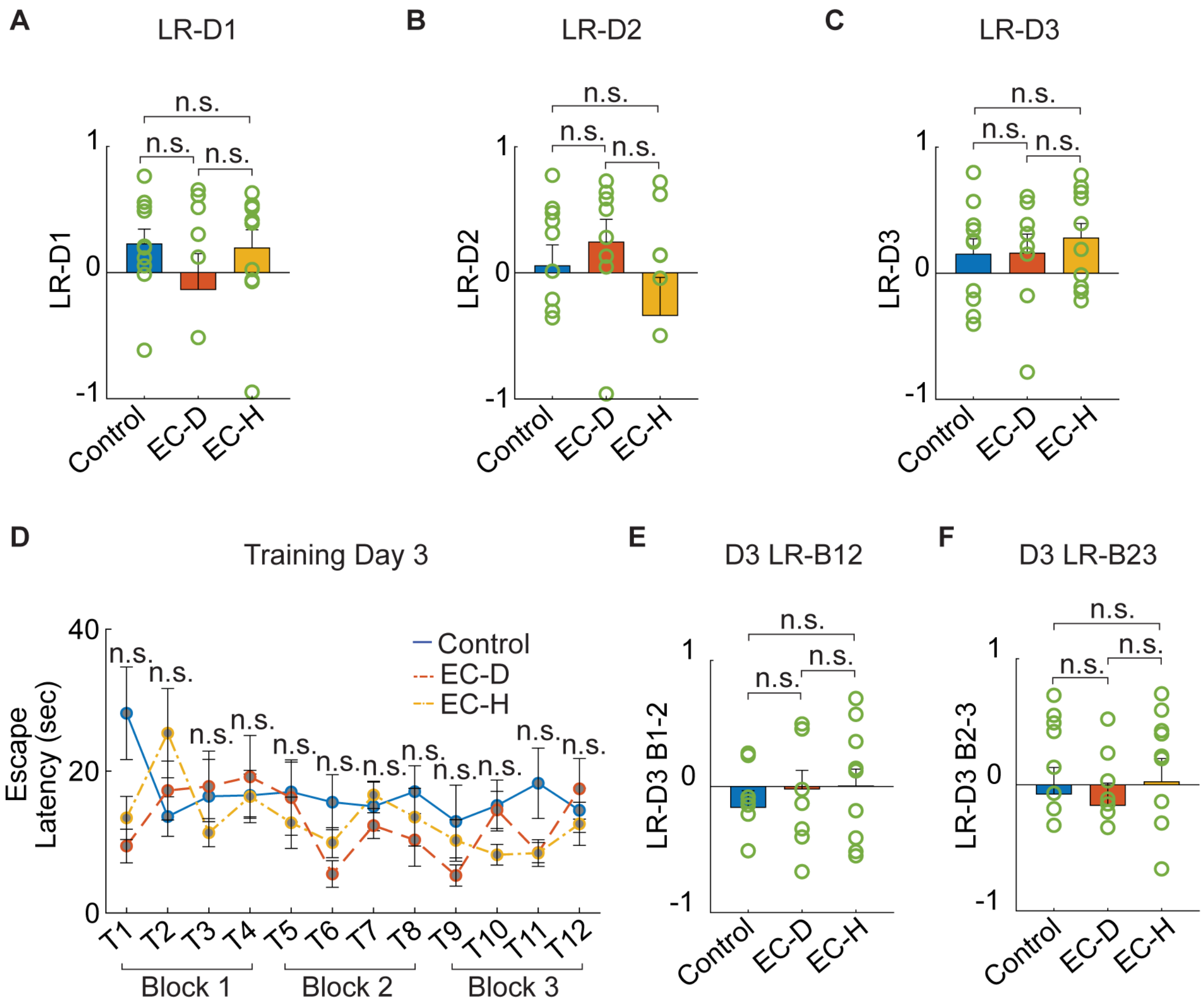

Figure S5. Chemogenetic hyperpolarization or depolarization of the TA pathway neurons did not affect the learning rate during training in the MWM task. (A) Summary bar graph showing the average learning rate on training day 1 (LR-D1), calculated from the escape latency from the first and the last block, of control (n = 10), EC-depolarization (EC-D) (n = 8), and EC-hyperpolarization (EC-H) (n = 10) mice. One-way ANOVA followed by Tukey's pairwise comparisons. Control vs. EC-D, p = 0.408; EC-D vs. EC-H, p = 0.473; Control vs. EC-H, p = 0.992. **(B)** Learning rate on training day 2 (LR-D2). One-way ANOVA followed by Tukey's pairwise comparisons. Control vs. EC-D, p = 0.856; EC-D vs. EC-H, p = 0.247; Control vs. EC-H, p = 0.478. **(C)** Learning rate on training day 3 (LR-D3). One-way ANOVA followed by Tukey's pairwise comparisons. Control vs. EC-D, p = 0.999; EC-D vs. EC-H, p = 0.815; Control vs. EC-H, p = 0.768. **(D)** Average escape latency of each trial on training day 3. Control mice (solid blue line), mice with EC-D (red dashed line), and mice with EC-H (orange dash-dot-dash line). Two-way mixed ANOVA followed by Holm-corrected post-hoc comparisons among the three groups for each trial on training day 3. Trial 1, Control vs. EC-D, p = 1; EC-D vs. EC-H, p = 1; Control vs. EC-H, p = 1; Trial 2, Control vs. EC-D, p = 1; EC-D vs. EC-H, p = 1; Control vs. EC-H, p = 1; Trial 3, Control vs. EC-D, p = 1; EC-D vs. EC-H, p = 1; Control vs. EC-H, p = 1; Trial 4, Control vs. EC-D, p = 1; EC-D vs. EC-H, p = 1; Control vs. EC-H, p = 1; Block 2: Trial 5, Control vs. EC-D, p = 1; EC-D vs. EC-H, p = 1; Control vs. EC-H, p = 1; Trial 6, Control vs. EC-D, p = 1; EC-D vs. EC-H, p = 1; Control vs. EC-H, p = 1; Trial 7, Control vs. EC-D, p = 1; EC-D vs. EC-H, p = 1; Control vs. EC-H, p = 1; Trial 8, Control vs. EC-D, p = 1; EC-D vs. EC-H, p = 1; Control vs. EC-H, p = 1; Block 3: Trial 9, Control vs. EC-D, p = 1; EC-D vs. EC-H, p = 1; Control vs. EC-H, p = 1; Trial 10, Control vs. EC-D, p = 1; EC-D vs. EC-H, p = 1; Control vs. EC-H, p = 1; Trial 11, Control vs. EC-D, p = 1; EC-D vs. EC-H, p = 1; Control vs. EC-H, p = 1; Trial 12, Control vs. EC-D, p = 1; EC-D vs. EC-H, p = 1; Control vs.

EC-H, p = 1. **(E)** Day 3 learning rate between block 1 and block 2. One-way ANOVA followed by Tukey's pairwise comparisons. Control vs. EC-D, p = 0.637, EC-D vs. EC-H, p = 0.996, Control vs. EC-H, p = 0.657. **(F)** Day 3 learning rate between block 2 and block 3. One-way ANOVA followed by Tukey's pairwise comparisons. Control vs. EC-D, p = 0.952; EC-D vs. EC-H, p = 0.806; Control vs. EC-H, p = 0.935. Data are represented as mean ± SEM. The reported sample sizes correspond to the number of mice. n.s. $p > 0.05$.
